# LSTM Neural Network for Inferring Conduction Velocity Distribution in Demyelinating Neuropathies

**DOI:** 10.3389/fneur.2021.699339

**Published:** 2021-07-01

**Authors:** Hiroyuki Nodera, Makoto Matsui

**Affiliations:** Department of Neurology, Kanazawa Medical University, Uchinada, Japan

**Keywords:** demyelination, conduction, deep learning, recurrent neural networks, nerve conduction studies

## Abstract

Waveform analysis of compound muscle action potential (CMAP) is important in the detailed analysis of conduction velocities of each axon as seen in temporal dispersion. This understanding is limited because conduction velocity distribution cannot be easily available from a CMAP waveform. Given the recent advent of artificial intelligence, this study aimed to assess whether conduction velocity (CV) distribution can be inferred from CMAP by the use of deep learning algorithms. Simulated CMAP waveforms were constructed from a single motor unit potential and randomly created CV histograms (*n* = 12,000). After training the data with various recurrent neural networks (RNNs), CV inference was tested by the network. Among simple RNNs, long short-term memory (LSTM) and gated recurrent unit, the best accuracy and loss profiles, were shown by two-layer bidirectional LSTM, with training and validation accuracies of 0.954 and 0.975, respectively. Training with the use of a recurrent neural network can accurately infer conduction velocity distribution in a wide variety of simulated demyelinating neuropathies. Using deep learning techniques, CV distribution can be assessed in a non-invasive manner.

## Introduction

A diagnosis of demyelinating neuropathy is significantly dependent on neurophysiological test results, as noted in the diagnostic criteria for chronic inflammatory demyelinating polyneuropathy, and Guillain–Barré syndrome (1, 2). Findings of nerve conduction studies, including conduction slowing, partial conduction block (CB), and abnormal temporal dispersion (TD), can suggest the presence of demyelinating neuropathy. Of those, TD is obtained by summating the peaks of opposite polarity generated by fast- and slow-conducting axons in a normal condition as well as peripheral neuropathy, (3) while its magnitude is pathologically augmented by greater degrees of conduction slowing caused by demyelination. Thus, the morphological evaluation of compound muscle action potential (CMAP) is critical for understanding underlying demyelination in terms of its degree, and variability in patients with demyelinating neuropathies.

A number of physiological studies have investigated conduction velocity (CV) distribution of individual axons. Methods used to directly measure CVs from individual axons include the near nerve technique, selective nerve fiber stimulation using special tungsten microelectrodes, F-wave-based testing, and collision-based testing (4). However, these techniques can be invasive and time-consuming, with a limited number of samples obtained, and are not widely utilized in clinical settings. Thus, methods based on the analysis of compound muscle or sensory action potential have been proposed. It is well known that compound motor action potential and sensory nerve action potential (SNAP) can be calculated from single fiber action potential by simply performing a summation of each component. It is considered that CV distribution can be measured if the inverse problem could be solved, namely, calculation of single fiber action potential from CMAP or SNAP. However, in many cases, the solution is not straightforward, as each single fiber action potential is not separable from others, and TD changes the morphology of CMAP and SNAP. A number of mathematical solutions have been proposed, such as for comparing two compound action potentials recorded from the same site with stimulation at different sites (4).

The recent advent of artificial intelligence has been utilized in various medical science areas. The results obtained in nerve conduction studies are regarded as time-series data, which are a series of data points ordered by time. In EEG and sleep studies, such time-series data have been utilized for a technique termed recurrent neural network, and its variations (5, 6). We speculated that by the use of artificial intelligence, especially deep learning, the above-mentioned “inverse problem” could be solved by deep neural networks, *i.e*., calculation of single fiber action potential from the compound action potential to obtain CV distribution of each axon.

The aim of the present study was to apply time-series analysis with deep learning to infer CV distribution from CMAP waveforms. To achieve this goal, we used a simulation study to obtain correlation data between CMAP waveforms, and CV distributions by providing large numbers of such paired data to be trained by deep neural networks.

## Methods

### Preparation of CMAP Waveforms

#### Preparation of Single Motor Unit Potential

A representative single motor unit potential (sMUP) was obtained from a published waveform recording obtained by stimulating the median nerve, and recorded from the abductor pollicis brevis (APB) of a healthy subject (7). Although the recording method was not specified in the manuscript, it was assumed to be incremental based on the description shown in the panel. The waveform was digitized by manual plotting using the freely obtainable software package Plot Digitizer (http://plotdigitizer.sourceforge.net/). A digitized sMUP waveform from 0 to 15 ms was then applied with a one-way cubic spline for data smoothing and curve-fitting (SRS1 Cubic Spline for Excel; SRS1 Software, LLC, Newton, MA, USA). After obtaining CMAP amplitudes at every 0.01 ms from the cubic spline, the amplitudes were set to 0 mV between 0–3.5 and 12.1–15 ms in order to not include stimulation artifacts and background noise for the summation of sMUP to organize CMAP. Data points obtained every 0.1 ms (0–15 ms, total of 150 time points) were then used for the preparation of CV distribution in order to reduce the amount of data.

#### Preparation for CV Distribution

The simulation was performed using the median nerve, with two stimulation sites at the wrist and elbow. The distance from the recording (APB) to the stimulation site at the wrist was arbitrarily set to 70 mm, and the distance between the two sites from the wrist to the elbow was 200 mm. For calculation purposes, conduction between the wrist and APB was considered to be normal, whereas various degrees of demyelination were present between the wrist and elbow. In both the distal and forearm segments, nerve conduction velocities were determined as, respectively uniform without focal conduction slowing in the short segment. In the case of CB, the CMAP amplitude was set to zero at all time points.

The distribution of CV as a histogram was obtained from a study performed by Elzenheimer et al. (8) who compiled clinical data from two studies that presented recordings from healthy subjects (9, 10) showing the relative frequencies for each 2 m/s velocity range. We digitized their published histogram to extract the number of axons at each interval from 38 to 64 m/s. The number of axons was set at 200 using a multiple motor unit number estimate technique previously reported (11). The results were used as a prototypical histogram of a normal nerve (Table 1).

**Table 1:** Distribution of conduction velocities in a normal model (8).

**Conduction velocity (m/s)**	**Number of axons (total 200)**
63	1
61	2
59	2
57	6
55	18
53	30
51	42
49	39
47	27
45	17
43	9
41	4
39	2
37	1

#### Preparation of Modeled Demyelination

In order to construct CMAP waveforms as seen in demyelination, two manipulations of the normal histogram created above were performed (Table 2). First, six patterns of CV slowing that applied to all the conducting axons were defined. Pattern 1 (“all range”) sets the CV of each axon at the same or slower than the original CV, with the slowest being 11 m/s. For example, the axon with the original CV of 53 m/s was randomly assigned a CV value of 53, 51, 49, …, 15, 13, or 11 m/s using a program written in Python. As for pattern 2 (“severe range”), each axon was randomly assigned 19, 17, 15, 13, or 11 m/s, while each in pattern 3 (“moderate range”) was randomly assigned 35, 33, 31, …, 25, 23, or 21 m/s. Each axon in pattern 4 (“mild range”) was assigned a value up to 8 m/s slower than the original CV value. For example, an axon at 53 m/s CV was assigned either 53, 51, 49, 47, or 45 m/s. For pattern 5 (“moderate range 2”), the axons were assigned a value up to 20 m/s slower. For example, an axon at 53 m/s CV was assigned 53, 51, 49, …., 37, 35, or 33 m/s. Axons in pattern 6 (“two distributions”) were assigned a value either up to 8 m/s slower or 11–19 m/s for severe slowing. For example, an axon at 53 m/s CV was assigned 53, 51, 49, 47, 45, 19, 17, 15, 13, or 11 m/s. The sets of CV slowing in these patterns did not have a CB; thus, they were listed as CB (–).

**Table 2:** Patterns of demyelinating models.

**Type**	**Method**
No conduction block [CB (–)]	
#1 (without CB1)	CV in entire range (unchanged or slower than the original CV)
#2 (without CB2)	CV slowing to severe range (11–19 m/s)
#3 (without CB3)	CV slowing to moderate range (21–35 m/s)
#4 (without CB4)	CV slowing to mild range (up to 8 m/s slower than the original CV)
#5 (without CB5)	CV slowing to moderate range 2 (up to 20 m/s slower than the original CV)
#6 (without CB6)	CV slowing in two distributions (up to 8 m/s or up to 11–19 m/s slower compared to the original CV)
With conduction block [CB (+)]	40% chance of conduction block in each axon
#1 (with CB1)	CV in entire range (unchanged or slower than the original CV)
#2 (with CB2)	CV slowing to severe range (11–19 m/s)
#3 (with CB3)	CV slowing to moderate range (21–35 m/s)
#4 (with CB4)	CV slowing to mild range (up to 8 m/s slower than the original CV)
#5 (with CB5)	CV slowing to moderate range 2 (up to 20 m/s slower than the original CV)
#6 (with CB6)	CV slowing in two distributions (up to 8 m/s or up to 11–19 m/s slower compared to the original CV)

*CV, conduction velocity*.

Next, data sets of CV distributions with CB were created. Six patterns of CV distribution similar to those noted above were separately created; then, some of the axons were assigned to an amplitude of zero throughout the duration to simulate CB. Each axon had a 40% chance of CB. These sets were listed as CB (+).

**Figure 1:**
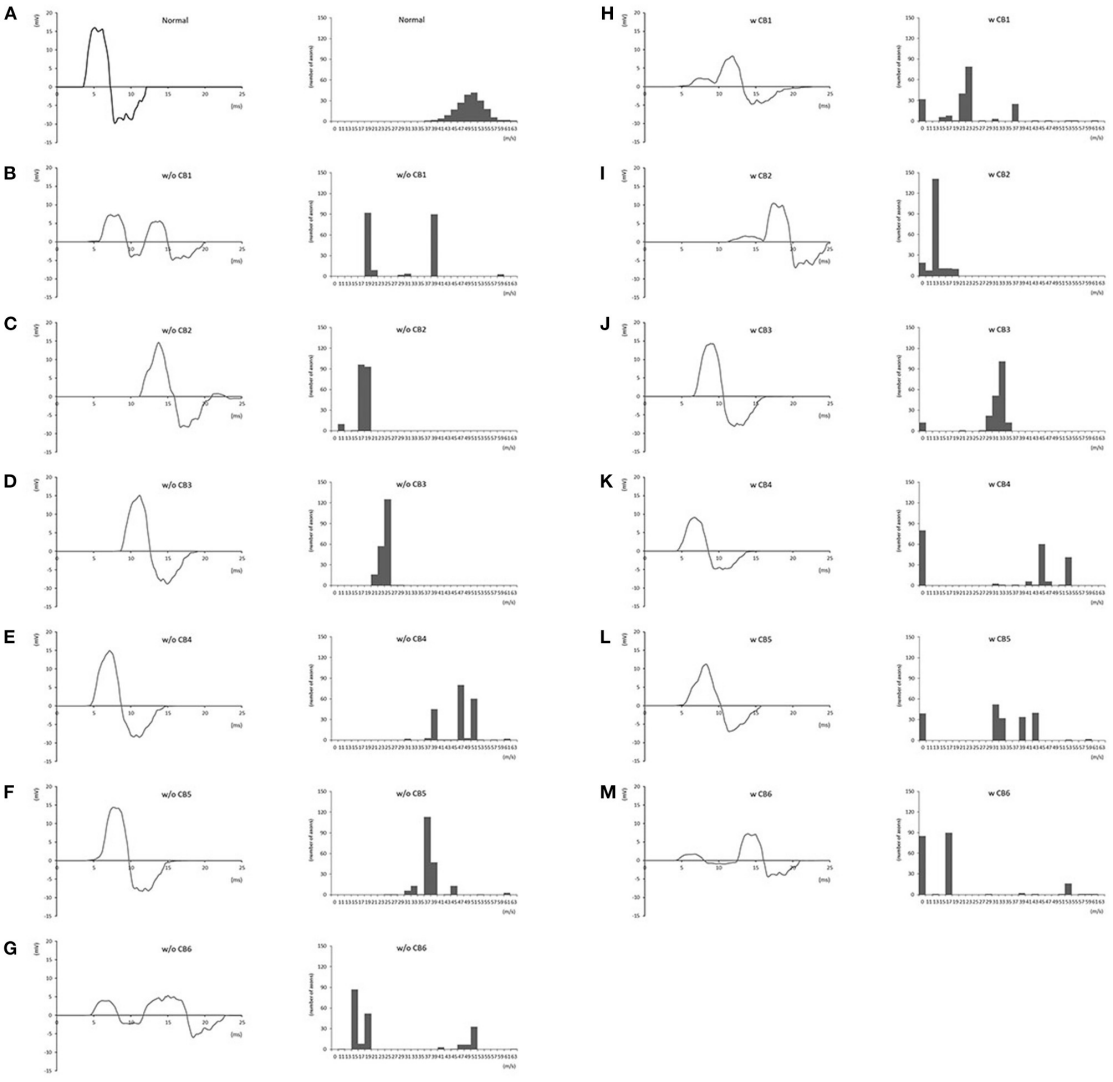
Representative compound muscle action potential waveforms and conduction velocity histograms for each group. **(A)**: normal; **(B)**: w/o CB1; **(C)**: w/o CB2; **(D)**: w/o CB3; **(E)**: w/o CB4; **(F)**: w/o CB5; **(G)**: w/o CB6; **(H)**: w CB1; **(I)**: w CB2; **(J)**: w CB3; **(K)**: w CB4; **(L)**: w CB5; **(M)**: w CB6 (see Table 2 for group definitions).

The master waveform of sMUP, created as noted in the section above, was then used to create CMAP waveform data by horizontally shifting the original waveform. For example, the original CV of 53 m/s in the fiber and the demyelinating CV of 45 m/s in the forearm were calculated to have distal latency based on the distance of 70 mm from the wrist to APB, while latency at the elbow was calculated based on 200 mm from wrist to elbow. After calculating and shifting each sMUP with the respective CV value, the sMUPs were superimposed to create the final CMAP data. According to the calculations, histograms and CMAP data were created for each group (1,000 each; total of 12,000). For use in training and testing by the deep neural network, the data were randomly split into training (80% of total, 9,600 sets) and test (20% of total, 2,400 sets) data. The obtained data were composed of 251 points (“waveform data”) of amplitudes (mV) of the final CMAP (0–25.0 ms), and the number of axons for the respective CV (“correct label”) (total number 28; 0, 11, 13, …, 61, and 63 m/s). Figure 1 shows the representative waveforms of CMAPs and velocity histograms.

#### Training and Testing by Deep Neural Network

**Figure 2:**
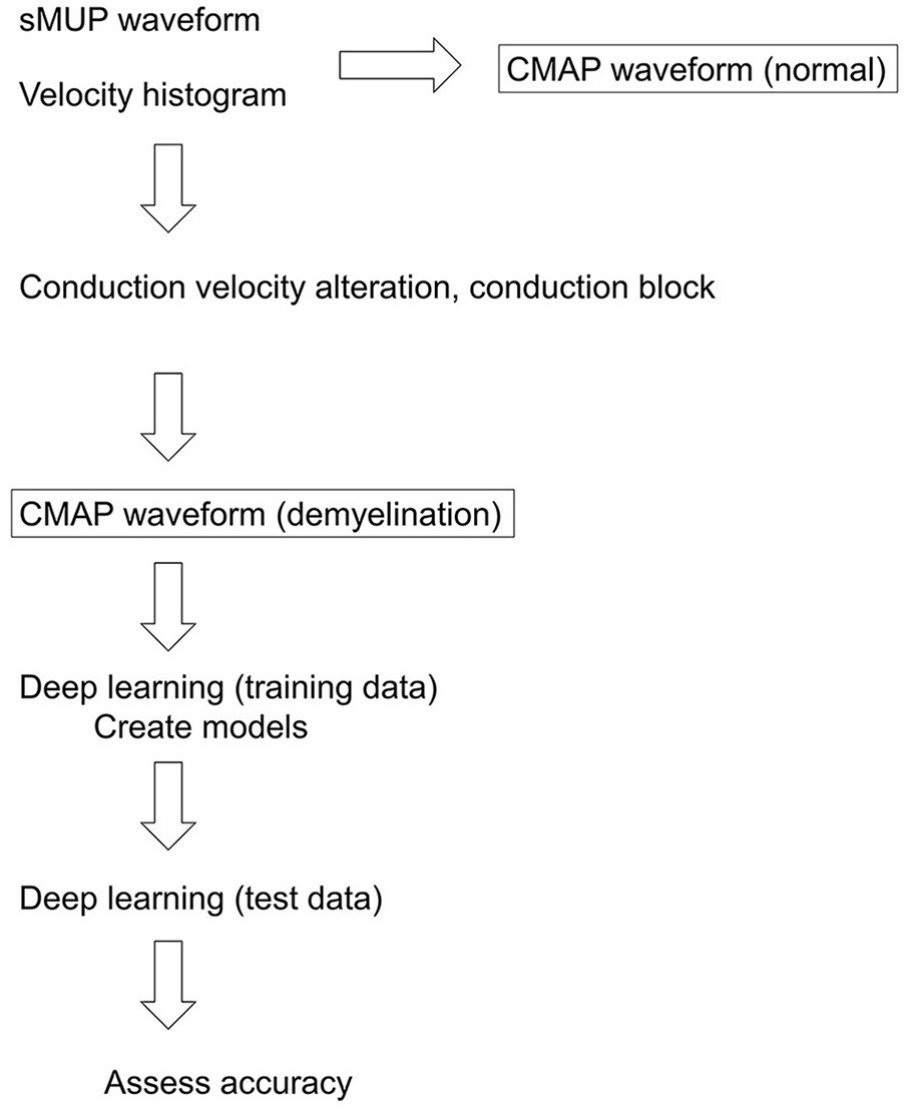
Overall flow of the study. sMUP, single motor unit potential; CMAP, compound muscle action potential.

Training and testing using a deep neural network were performed with TensorFlow 2.4. Data prepared as described above were fed into deep neural networks. The sequential Keras model was used, with the following parameters: epoch, 1,000; dropout rate, 0.5; number of hidden layers, 1,000; batch size, 2,048; loss, mean squared error; optimizer, adam; metrics, accuracy; early stopping, monitor “loss”; patience, 2. The following recurrent networks were also employed using the standard Keras functions: simple recurrent neural network (simple RNN), long short-term memory (LSTM), gated recurrent unit (GRU), bidirectional LSTM, and bidirectional GRU. Additionally, the number of layers was changed (one, two, or three) for LSTM, bidirectional LSTM, GRU, and bidirectional GRU. For prediction, the number of the axons at each CV was rounded off and compared with the correct label. A summary of the overall process is presented in Figure 2.

## Results

**Figure 3:**
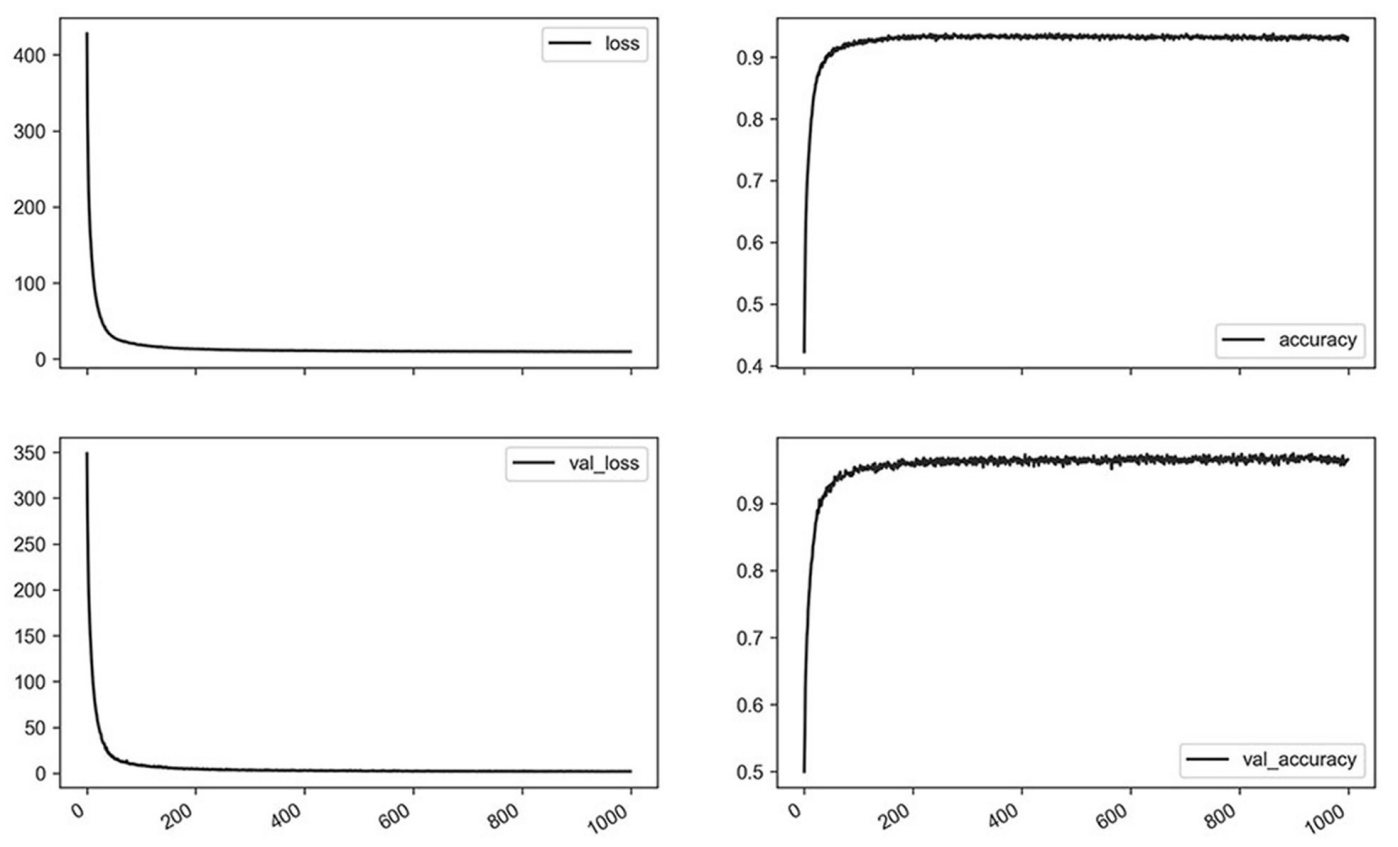
Losses and accuracy of training for the representative network (bidirectional long short-term memory, two layers, 1,000 epochs) using a whole dataset.

Representative curves for loss and accuracy are shown in Figure 3, both of which showed a steady decrement or increment without developing overfitting. The training results summarized by each network are presented in Table 3. Overall, the LSTM network showed a higher accuracy and fewer losses than simple RNN, while GRU had a slightly lower accuracy, and more losses than LSTM. Furthermore, bidirectional LSTM showed a higher accuracy and fewer losses as compared to unidirectional LSTM. Regarding the number of recurrent layers, two layers showed the highest accuracy and the fewest losses. In summary, the best results were obtained with two-layer bidirectional LSTM, which showed a training accuracy of 0.954.

**Table 3:** Network comparison (1,000 epochs).

	**Number of layers**	**Training loss**	**Training accuracy**	**Validation loss**	**Validation accuracy**
Simple recurrent neural network	1	23.043	0.929	8.387	0.930
Long short-term memory (LSTM; unidirectional)	1	16.572	0.933	3.225	0.965
	2	3.419	0.937	1.519	0.976
	3	5.002	0.922	2.137	0.959
Bidirectional LSTM	1	9.603	0.930	2.201	0.963
	2	1.831	0.954	0.752	0.975
	3	3.115	0.943	1.130	0.974
Gated recurrent unit (GRU; unidirectional)	1	15.722	0.925	3.026	0.958
	2	3.077	0.938	1.430	0.967
	3	5.482	0.919	2.120	0.962
Bidirectional GRU	1	9.087	0.933	1.829	0.966
	2	1.988	0.955	0.783	0.975
	3	3.349	0.939	1.33	0.972

Next, in order to identify accuracy and loss by the various simulation sets, those in the respective subsets were calculated (Table 4). Overall, subsets with no CB tended to show a higher accuracy and fewer losses than those with a CB. There were variations regarding accuracy and loss found in each dataset, with subtype pattern 4 (mild range conduction slowing with or without CB) showing the lowest accuracy and greatest number of losses.

**Table 4:** Results with each dataset [bidirectional long short-term memory (LSTM) with two LSTM layers, 1,000 epochs].

**Conduction velocity (CV) slowing type**	**Conduction block**	**Training loss**	**Training accuracy**	**Validation loss**	**Validation accuracy**
1 (without CB1)	Absent	0.141	0.983	0.109	0.980
2 (without CB2)		0.082	0.991	0.031	1.000
3 (without CB3)		0.223	0.983	0.090	0.970
4 (without CB4)		3.786	0.956	2.752	0.945
5 (without CB5)		0.462	0.978	0.160	0.990
6 (without CB6)		0.218	0.988	0.114	0.990
1 (with CB1)	Present	0.187	0.974	0.105	0.985
2 (with CB2)		0.211	0.980	0.096	1.000
3 (with CB3)		0.352	0.965	0.092	0.985
4 (with CB4)		1.789	0.976	0.823	0.985
5 (with CB5)		0.719	0.964	0.321	0.960
6 (with CB6)		0.244	0.984	0.092	0.985

*(1), conduction velocity slower in all ranges (original velocity 11 m/s)*.

*(2), CV slowing to 11–19 m/s*.

*(3), CV slowing to 21–35 m/s*.

*(4), CV up to 8 m/s slower than the original velocity*.

*(5), CV up to 20 m/s slower than the original velocity*.

*(6), CV up to 8 or 11–19 m/s slower than the original velocity*.

**Figure 4:**
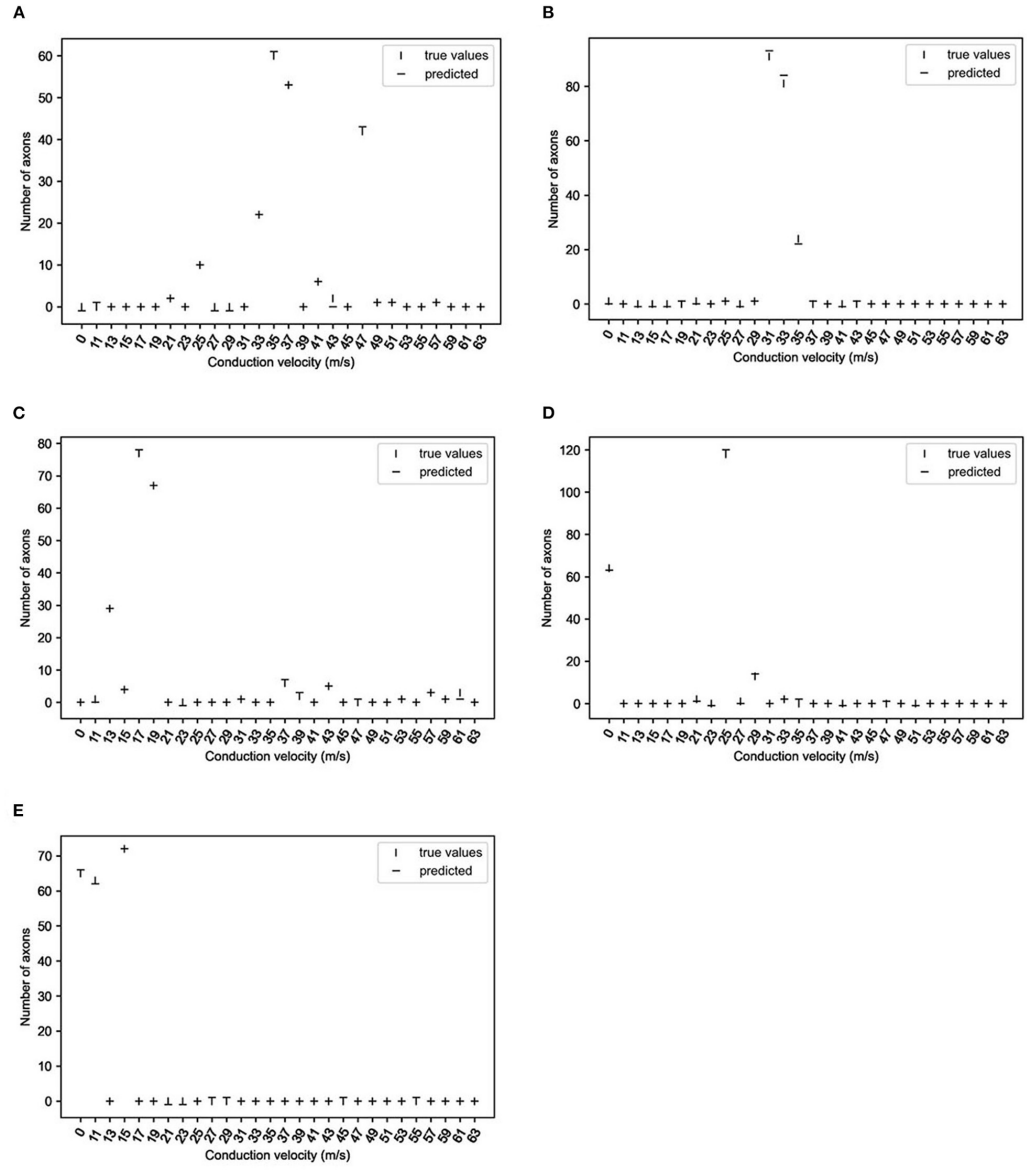
True and predicted values of representative data (two-layer bidirectional long short-term memory; 10,000 epochs). The vertical lines show the number of axons at the respective conduction velocities, and horizontal lines predicted the numbers of axons. **(A)**: without CB1; **(B)**: without CB3; **(C)**: without CB6; **(D)**: with CB3; **(E)**: with CB6 (see Table 2 for group definitions).

Finally, the network with the best accuracy/loss profile (*i.e*., two-layer bidirectional LSTM) was used to infer test data. As shown in Figure 4, those velocity histograms satisfactorily predicted true values regardless of the absence or presence of a CB or CV range.

## Discussion

In this study, deep learning of simulated CMAP waveforms was performed to infer CV distribution. The use of a two-layer bidirectional LSTM network showed the best results for accuracy and loss. As a result, the simulated CV distributions were satisfactorily inferred by the deep learning network.

### Application of Recurrent Neural Networks to CMAPs

CMAPs are composed of time-series signals that change their amplitude over time, and various methods have been used to process and analyze time-series signals. The earlier proposed time-series analysis systems were largely based on handcrafted techniques, which were followed by systems based on feature extraction and machine learning. That trend showed a further advancement with the recognition of deep neural networks as powerful tools to consider various type of signals, including images and time-series signals.

Neural signals are a good example of time-series signals and have been analyzed using the methods noted above (12). On the other hand, CMAPs have not been studied with the use of deep learning-based methodologies, presumably because of their straightforward signal characteristics and relative lack of morphological complexity. However, once a CMAP is taken as the summation of a few 100 composite waves of single MUPs, the situation dramatically changes and a CMAP becomes a complex target to analyze.

An RNN is a type of artificial neural network that uses sequential or time-series data. These networks are designed to recognize sequential characteristics in data and use patterns for prediction and thus are used in signals and languages. To improve RNN so as to avoid the vanishing gradient and exploding gradient problems, LSTM became utilized for longer data sequences. Thereafter, GRU was introduced as an alternative of LSTM to reduce the number of calculations. Bidirectional LSTM and bidirectional GRU are sequence processing models that consist of two LSTMs or GRUs, one that receives input in a forward direction and the other in a reverse direction. Another advancement has been provided by use of multiple layers of recurrent networks. Such complex neural network structures were used to analyze CMAPs in the present study, and they showed the superiority of bidirectional networks as compared to their unidirectional counterparts with both LSTM and GRU. On the other hand, the use of multiple layers gave mixed results, as two layers showed better results than a single layer, while a three-layer network had a decreased accuracy. That decrease with a complex neural network structure would be due to overfitting.

### Non-Invasive Assessment of CV Distribution

Rhee et al. (13) studied the effects of phase cancellation on the amplitudes of CMAPs with computer simulation in an animal study, and showed the significance of the fastest conducting fibers for determining the amplitude and area of a CMAP. Later, Van Asseldonk et al. (14) proposed novel diagnostic criteria for CB and TD in the forearm segment of the median nerve using simulations with the surface-recorded MUAPs. Recently, those early results presented by Rhee et al. were challenged by Elzenheimer et al. (8), as they noted that the CMAPs were obtained in that study by weighted summation of only a single-representative sMUP from an animal study that showed an apparently different sMUP morphology from that seen in humans. Other studies have conducted measurements of slower-conducting fibers with a collision technique and the use of single fiber electromyography (15, 16), though those techniques are time consuming, and not readily available for daily clinical practice. To the best of our knowledge, no previous report has presented findings obtained by non-invasive inference of CV distribution using a deep learning method.

### Limitations

This study has some limitations, including the simulation-based design with a single sMUP used to represent the remaining sMUPs. Keenan et al. (17) examined the influence of motor unit properties on the size of simulated evoked surface EMG potential and reported that ~7% of the motor unit potentials were responsible for 50% of the size of the evoked potential. The CMAP in the present study was obtained by the summation of 200 motor units with identical morphology. More precise reproduction would be available by simulating with a group of non-uniform sMUPs, which was also recently proposed by another group (8). Furthermore, the CMAP did not cover a potentially observable morphology in an actual nerve conduction study. With the present method, we initially randomized the CV distribution rather equally to all potential candidates, which resulted in a similar CV distribution and CMAP morphology, and randomization was performed with a significant weight shift to create variable waveforms. We admit that neurophysiological estimate has some limitations. One good example is motor unit number estimate that is based on the paradigm that a summated value for the total motor unit population within a nerve is divided by a value representing the average sMUP to yield an estimate. Similar to motor unit number estimate, the velocity estimate in the present study has an inherent limitation to the accuracy of the results in comparison to the ground truth that is not easily available. On the other hand, both methods should be useful in the longitudinal comparison of a single subject in order to monitor the clinical improvement or decline.

Finally, this study was limited to the forearm segment of the median nerve, and other nerve segments should be separately investigated. The conduction features characteristic for each nerve segment should also be taken into consideration, such as the effects of phase cancellation and far-field potentials (18, 19). The present results can be applicable to other nerves and/or other nerve segments by altering the parameters, such as the number of axons, inter-stimulating distance, and velocity histogram. We admit that the present study was a proof-of-concept project which was indeed successful from the authors' perspective. We applied the model to a recorded CMAP waveform in a patient with neuropathy. However, the inference of the CV estimate was not comparable with the truth which cannot be made available from a live patient. One rough estimate would be measurement with a single-fiber EMG, but only part of axon conduction can be assessed by the method.

### Clinical Significance

The waveform analysis of CMAPs is an important process to elucidate the underlying pathological features for care of patients with demyelinating neuropathies, and fascicle-to-fascicle variation of conduction velocities implies their presence. Tuncer et al. (20) reported an animal diabetic model that showed CV distribution abnormality earlier as compared to the conventional measures of a nerve conduction study. Furthermore, a detailed knowledge of the conduction of each axon would be useful for the serial evaluation of affected patients as that could identify interval clinical changes. We consider that the non-invasive inference of CV distribution and frequency of CB should be recognized as a “non-invasive nerve biopsy” procedure, as important information can be obtained.

Of note is the fact that training the model requires computer resources, including graphics processing units. Inference is, on the contrary, straightforward and available with a less powerful computer or even a smart phone. Therefore, the technology would be easily available with a regular EMG machine or a smart phone.

## Data Availability Statement

The raw data supporting the conclusions of this article will be made available by the authors, without undue reservation.

## Ethics Statement

This study was approved by the research ethics committee of Kanazawa Medical University.

## Author Contributions

HN and MM contributed to conception and design of the study. HN organized the database, performed the analysis, and wrote the first draft of the manuscript. Both authors contributed to the manuscript revision read and approved the submitted version.

## Conflict of Interest

The authors declare that the research was conducted in the absence of any commercial or financial relationships that could be construed as a potential conflict of interest.

## Abbreviations

AI: artificial intelligence
APB: abductor pollicis brevis
CB: conduction block
GRU: gated recurrent unit
LSTM: long short-term memory
MUNE: motor unit number estimate
NCS: nerve conduction study
RNN: recurrent neural network
TD: temporal dispersion.
